# RNA-binding proteins in bone pathophysiology

**DOI:** 10.3389/fcell.2024.1412268

**Published:** 2024-06-20

**Authors:** Paola Maroni, Noemi Anna Pesce, Giovanni Lombardi

**Affiliations:** ^1^ Laboratory of Experimental Biochemistry and Molecular Biology, IRCCS Istituto Ortopedico Galeazzi, Milano, Italy; ^2^ Department of Athletics, Strength and Conditioning, Poznań University of Physical Education, Poznań, Poland

**Keywords:** RNA binding proteins (RBPs), bone pathophysiology, post-transcriptional regulatory mechanisms, modulation of mRNAs, osteoporosis, bone aging, bone cancer, bone metastasis

## Abstract

Bone remodelling is a highly regulated process that maintains mineral homeostasis and preserves bone integrity. During this process, intricate communication among all bone cells is required. Indeed, adapt to changing functional situations in the bone, the resorption activity of osteoclasts is tightly balanced with the bone formation activity of osteoblasts. Recent studies have reported that RNA Binding Proteins (RBPs) are involved in bone cell activity regulation. RBPs are critical effectors of gene expression and essential regulators of cell fate decision, due to their ability to bind and regulate the activity of cellular RNAs. Thus, a better understanding of these regulation mechanisms at molecular and cellular levels could generate new knowledge on the pathophysiologic conditions of bone. In this Review, we provide an overview of the basic properties and functions of selected RBPs, focusing on their physiological and pathological roles in the bone.

## 1 Introduction

### 1.1 Basic principles in bone biology

**FIGURE 1:**
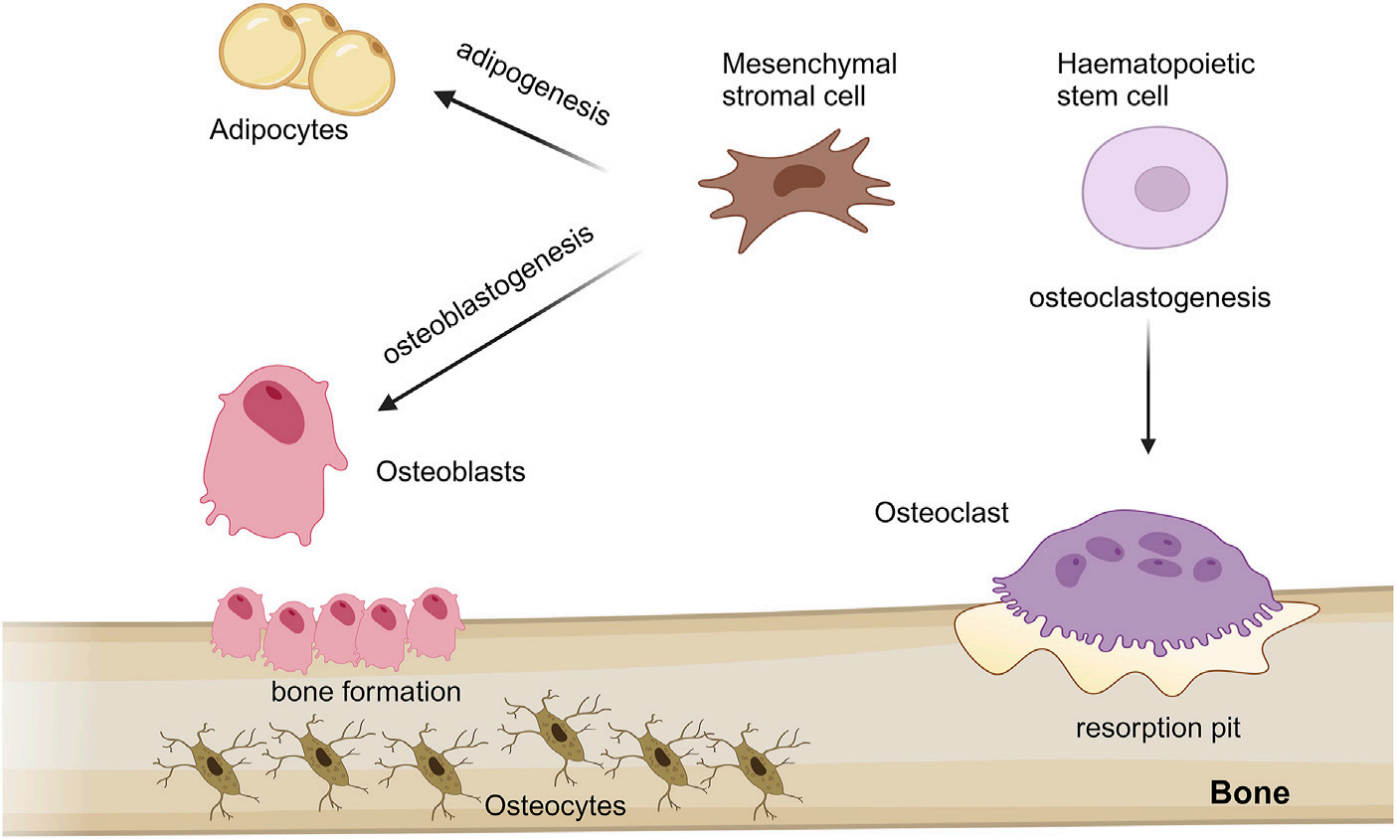
Schematic representation of mesenchymal stem cells and haematopoietic stem cells differentiation processes into different cell type in bone. Mesenchymal stromal cells (MSCs) predominantly proliferate and differentiate into bone-forming osteoblasts and adipocytes, throught osteoblastogenesis and adipogenesis processes, respectively. During aging, adipogenesis can accelerate compared to osteoblastogenesis, thus potentially leading to osteoporosis and bone loss. MSCs give rise also osteocytes, which are osteoblasts trapped in the bone matrix; while, osteoclasts, implicated in bone resorption, originate from haematopoietic stem cells through a process known as osteoclastogenesis. The figure was created with http://biorender.com.

Bone is a highly dynamic mineralized connective tissue responsible for supporting the body, protecting internal organs, producing blood cells, storing calcium and fat tissue (Weatherholt et al., 2012) and, as more recently depicted, having endocrine functions (Kirk et al., 2020). Bone tissue, like all connective tissues, contains relatively few cells and large amounts of extracellular matrix (ECM). The bone matrix comprises organic (collagenous and non-collagenous proteins, proteoglycans, γ-carboxyglutamic acid-containing proteins, glycoproteins and small integrin-binding ligand N-linked glycoproteins/SIBLINGs) and inorganic compounds (hydroxyapatite) (McKee et al., 2019; Lin et al., 2020). Although bone cells account for less than 2% of the entire bone mass, they are crucial to bone functions. Four types of cells constitute bone tissue: osteoblasts, osteocytes, osteoclasts and mesenchymal stromal cells (MSCs). All these cells strongly cooperate to prevent the accumulation of damage and maintain the mechanical strength (Mohamed, 2008). Osteoblasts originate from MSCs and, in the adult skeleton, represent 4%–6% of the total bone cells; they are implicated in bone formation, through the synthesis and the secretion of collagen matrix and calcium salts. Osteocytes represent the most abundant cells (90%–95% of total bone cells) and derive from osteoblasts through a multiphase process of differentiation; their principal role is mechanosensing. Otherwise, osteoclasts that originate from haematopoietic stem cells with the function of resorbing bone. Regularly, osteoclasts break down old bone, while osteoblasts form new bone; the balance between both activities is responsible for the constant remodelling of bone (Figure 1) (Sromova et al., 2023).

MSCs are multipotent stromal cells capable of self-renewing and differentiating into multiple cell lineages, including osteoblasts and adipocytes, through a process known as osteoblastogenesis and adipogenesis, respectively (Ullah et al., 2015). Adipocytes, the cellular unit of adipose tissue, are specialized in storing energy as fat. MSC commitment is linked to the activation of specific cell signalling pathways, which simultaneously lead to differentiated cell type and suppression of competitive lineages (Chen et al., 2016; Muruganandan et al., 2020). An impairment of this finely tuned mechanism can lead to an imbalance of adipogenesis-osteoblastogenesis, which can be relevant for the development of different bone diseases, including osteoporosis and bone loss (Figure 1) (Savopoulos et al., 2011).

## 2 RBPs and their characteristics

RNA-binding proteins (RBPs) are a class of proteins responsible for the regulation of post-transcriptional events in the cells, influencing mRNA fate and final protein levels (Gerstberger et al., 2014; Engel et al., 2020). Indeed, RBPs play crucial roles in various aspects of RNA metabolism influencing transcription, splicing, nuclear export, stabilization, degradation, translation, localization and are critically involved on translation or repression of transduction. Structurally, classic RBPs are identified by the presence of one or more multiple binding specificities highly represented in the genome, known as RNA-binding domains (RBDs), responsible for targeting sequence motifs of RNAs and partner proteins. Generally, the recognition sequences of RBDs are extremely short (<100 residues), thus their capacity to interact with RNAs is restricted. However, two or more RBDs can be bound together to create a larger binding interface that recognizes a longer sequence (Neelamraju et al., 2015; Marchese et al., 2016).

Prevalently, the RBDs include the RNA recognition motif (RRM), the most conserved region in higher vertebrates, which consists of about 70–90 aminoacids (aa). Generally, RRM contains two highly conserved peptide motifs, namely, an octapeptide and a hexapeptide, characterized by three conserved Arginine/Lysine residues that are crucial for the interactions with the nucleobases of RNAs (Manival et al., 2001; Maris et al., 2005). RBDs can include also the K-homology domain (KH), which contains 60 aa with a characteristic of hydrophobic residues that make hydrogen bonds necessary for binding targets. Other sequences can also characterize RBDs, such as the C3H1 zinc-finger domain, (ZF) double-stranded RBDs and various others, all of which are needed to bind target sequences through molecular interactions of chemical moieties, addressing the functional requirements of RBPs (Gerstberger et al., 2014; Dominguez et al., 2018).

Additionally, by a combination of various RBDs, RBPs can interact with different regions of targets; the composition of RNA interactomes is environment-dependent and responds to specific stimuli (Feng et al., 2019; Corley et al., 2020).

### 2.1 Function of RBPs

RBPs bind single or double-stranded RNAs, influencing their fate from synthesis to decay. RBPs are implicated in regulating post-transcriptional processes, such as splicing, processing, transport, translation and modulation of mRNAs (Kelaini et al., 2021).

**FIGURE 2:**
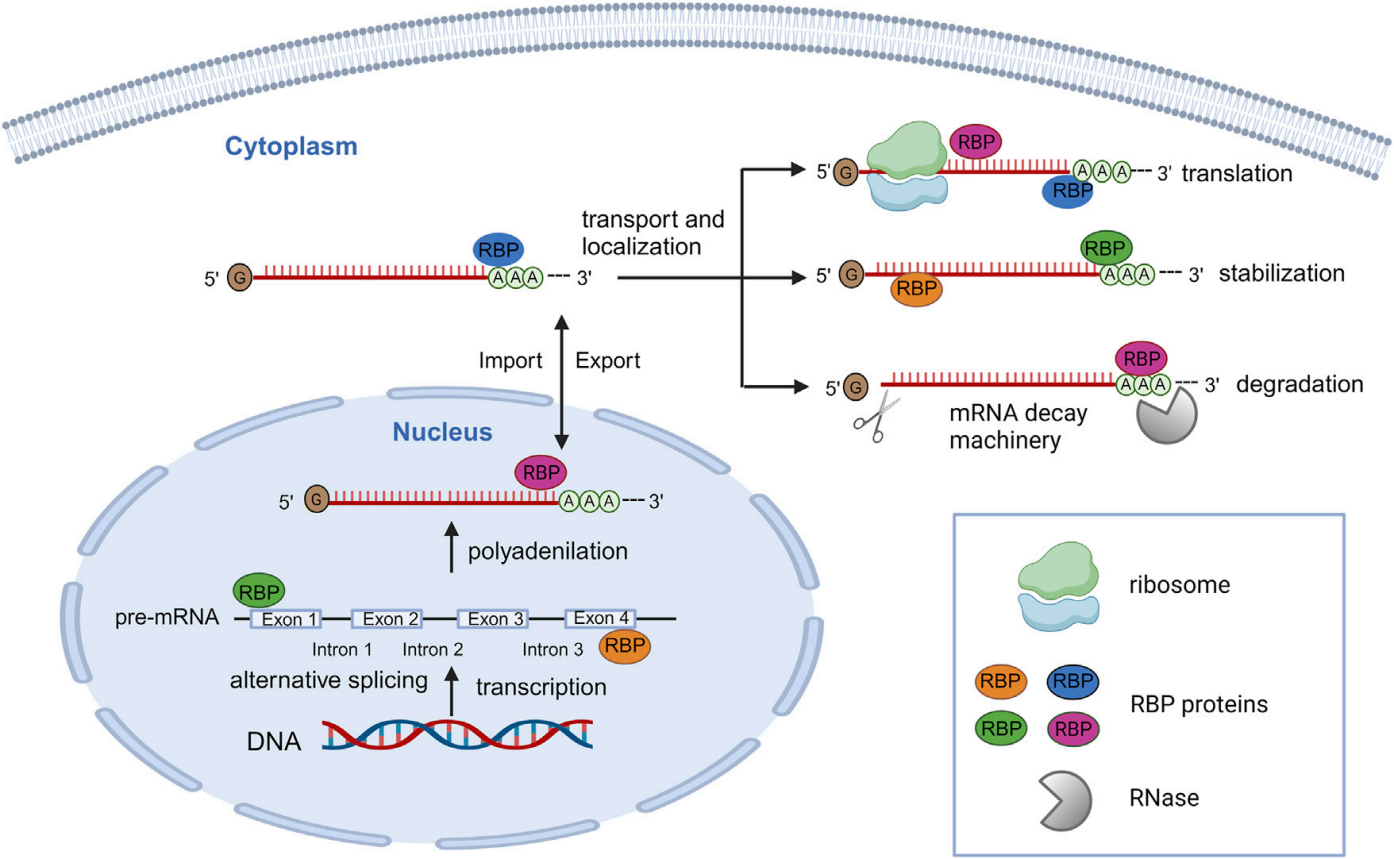
Schematic representation of post-transcriptionally regulation of gene expression by a large number of RBPs. In the nucleus, RBPs can bind pre-mRNAs, regulating alternative splicing, polyadenylation and allowing their export. In the cytoplasm, RBPs can interact with mRNAs for regulating their localization, stabilization, degradation, and translation activation/repression. The figure was created with http://biorender.com.

Crucially for RNA-related processes, RBPs are found in both the nucleus and cytoplasm and, their localization, dynamically changes based on cellular context and stress conditions. For instance, in the nucleus RBPs can facilitate alternative splicing of mRNAs by binding their coding region, while in the cytoplasm, RBPs can regulate mRNA localization or can induce mRNA degradation or stabilization through the binding with their 3′ untranslated region (UTR) domain (Figure 2) (Hentze et al., 2018).

Recently, a role for RBPs has been recognized to take part in extracellular vesicles (EVs) action: individual RBPs were detected in EVs along with their RNA substrates and RBPs emerged as key players in the sorting of specific miRNAs into EVs influencing the composition of vesicle cargo (Fabbiano et al., 2020). Certain RBPs are involved in recognizing specific RNA motifs or structures that guide the sorting process. Moreover, EVs, released from one cell, can deliver not only the RNA molecules but also the associated RBPs. The interaction of RBPs and RNA molecules within EVs may affect the fate of the vesicles cargo in the extracellular space (Statello et al., 2018). This role of RBPs in the entrapment of RNA species into EVs underlines that the interplay between RBPs and EVs contributes to the complexity of RNA-mediated cellular processes and cell-to-cell intercellular communication and has implications for normal cellular functions as well as disease processes.

Given the important role of RBPs in maintaining cellular homeostasis under physiological conditions, any alteration in RNA-RBP interactions and any change in RBP expression or function can lead to the development of various diseases, including diabetes, neurodegenerative diseases, but also cancer and bone-related diseases.

#### 2.1.1 Role of RBPs in the nucleus

Heterogeneous nuclear ribonucleoparticles (hnRNPs) have been identified as a large family of RBPs that interact with RNAs in the nucleus to form ribonucleoprotein complexes, thus mediating transcription control and nuclear processing of transcripts, including alternative splicing and polyadenylation (Witten and Ule, 2011; Wippel et al., 2021).

In eukaryotic, alternative splicing guarantees the generation of different pre-RNAs from the same gene, contributing to both transcriptome and proteome complexity (Wang et al., 2015; Tao et al., 2024). At molecular level, this process is guaranteed by an important macromolecular complex, the spliceosome, and a combination of several proteins, which cooperate to regulate a series of RNA-RNA and RNA-protein interactions (Tao et al., 2024). In general, by binding regulatory sequence elements of pre-mRNAs, hnRNP can act as either an activator or repressor of spliceosome recruitment, promoting or suppressing the inclusion of an exon, thus contributing to the generation of different mRNA transcripts (Horn et al., 2023). hnRNPs-preRNA binding can occur in both intronic and exonic domains, and influences the regulatory mechanisms of mRNA and its fate in the cytoplasm (Garcia-Maurino et al., 2017) (Figure 1).

Commonly, hnRNPs play multifunctional roles in each step of the RNA life cycle, hence, hnRNPs implicated in alternative splicing are also involved in polyadenylation (Huang and Carmichael, 1996). Polyadenylation, which consists in the addition of multiple adenosine (A) monophosphates at the 3′ ends of preRNA transcripts, enhances the translation of an mRNAs and accomplishes via coordinative actions of several molecules that include hnRNPs (Glisovic et al., 2008; Proudfoot, 2011). hnRNPs have been shown to regulate polyadenylation events, by competing or reinforcing the binding of the polyadenylation machinery proteins to their target sites. In this manner, hnRNPs indirectly contribute to guarantee a further generation of different mRNA isoforms either allowing or denying mRNAs nuclear transportation, their translation efficiency and their stability (Erson-Bensan, 2016; Stewart, 2019).

Several evidences have reported hnRNPs as shuttle between nucleus and cytoplasm (Michael et al., 1995; Visa et al., 1996), suggesting a possible role of these proteins in mature mRNA. hnRNP A1 is one of the most abundant proteins in the nucleus, localized to the nucleoplasm, that has a role in nuclear import-export (Siomi and Dreyfuss, 1995; Izaurralde et al., 1997). In mammalian cells, hnRNP A1 levels have been shown to be in constant flux between the nucleus and the cytoplasm (Biamonti et al., 1993). In fact, hnRNP A1 leaves the nucleus with mRNAs, dissociates from the mRNA in the cytoplasm and is then re-imported into the nucleus. Shuttling is not passive, hnRNP A1 contains nuclear export signals, determined by a 38-amino acid sequence located near the COOH terminus, which is essential to mediate nuclear export of the protein in the absence of RNA binding (Michael et al., 1995).

#### 2.1.2 Role of RBPs in the cytoplasm

Different cellular compartments provide different chemical environments and various potentially interacting partners for RBPs. In the cytoplasm, RBPs have been shown to be involved in mRNAs translation, localization and stability (Yan et al., 2021). Furthermore, RBPs can interact with UTRs or specific RNA and protein sequences to regulate cellular responses following different environmental stresses, such as nutrient deprivation and viral infection, or to modulate cell proliferation, differentiation or cell death in healthy organisms (Spriggs et al., 2010; Walsh and Mohr, 2011; Martinez-Salas et al., 2013).

During protein synthesis, the sequences of an mRNA molecule are converted into amino acid sequences, through a high coordination of different proteins and RNAs that takes place in macromolecular machines called ribosomes. Generally, RBPs have been shown to interact with 5′ and 3′UTRs and coding regions of mRNAs, expressing regulatory functions to modulate ribosome recruitment and transit and, in most cases, providing the landing site of other RBPs (Sonenberg and Hinnebusch, 2009; Harvey et al., 2018). Several evidences (Tarun and Sachs, 1995) derived from genetic and biochemical experiments in yeast, have shown that a class of RBPs, called poly(A) binding proteins (PABPs), is essential to the initiation of translation of mRNA by binding the poly(A) tail. Through a direct interaction with translation initiation factor 4E (eIF4E), PABPs increase translation initiation efficiency and prevent de-adenylation and degradation events on transcripts. In line with this, experiments on mammalian cells have shown that the lack of PABPs reduces rates of translation, decreases the efficiency of the initiation complex formation, and hampers the interaction between eIF4E-mRNAs, thus demonstrating the essential role of PABPs during mRNAs translation events (Kahvejian et al., 2005).

mRNAs translation is tightly coupled to mRNA turnover; RBPs are involved in this mechanism, in order to regulate mRNA stability and maintain protein homeostasis (Chan et al., 2018). Indeed, RBPs are involved in the regulation of mRNAs cleavage and/or translational repression, by binding to mRNAs sequence elements rich in adenosine (A) and uridine (U), called AU-rich elements (AREs), which are typically found in the mRNA 3′UTR. RBP-ARE binding causes the recruitment of other effectors, such as mRNA decay machinery and protein kinases, to target mRNAs, thus promoting destabilization/degradation of mRNAs that might be involved in various biological functions such as proliferation, differentiation, signal transduction, and metabolism (Otsuka et al., 2019).

This review provides an overview of the basic properties and functions of some RBPs and focuses on the physiological and pathological roles of RBPs closely related to bone. The articles included in this review were retrieved from PubMed by combining the following terms: RNA-binding proteins, RBPs, bone pathologies, bone cells, osteoclasts, osteoblasts, RBDs, hnRNPs, RNAs regulation, RBPs in bone cell proliferation, RBPs as target, bone metastasis, bone cancer, osteoporosis, Ewing sarcoma, osteosarcoma, miRNAs, therapeutic strategy and therapeutic targets.

## 3 Physiological roles of RBPs in bone cells

During skeletal development, to promote a net gain in bone mass, the anabolic activity of osteoblasts is favoured over the catabolic activity of osteoclasts (Salhotra et al., 2020). Conversely, at bone maturity, in order to maintain the bone mass, bone remodelling expects a balance between the activity of both osteoblasts and osteoclasts. In both phases, this process requires a tight synchrony between all bone cells and a strict regulation by different biochemical factors (Cong et al., 2019). In bone cells, RBPs are crucial during cellular commitment, differentiation, proliferation and senescence (Park et al., 2020; Zhao et al., 2022).

### 3.1 Msi2

Musashi2 (Msi2) is a member of an evolutionarily conserved family of RBPs that is implicated in the regulation of post-transcriptional events in the cells, yet proliferation and differentiation of stem cells by binding specific mRNAs. Msi2 is known to be a key regulator in cancer initiation and progression (Duggimpudi et al., 2018). However, it has been shown to be involved in the regulation of MSCs’ fate commitment and bone homeostasis (Park et al., 2014). Experiments of cultured bone marrow mesenchymal stromal cells (BMSCs) from both WT and *Msi2* ^*−/−*^ mice showed that Msi2 enhances osteoblast differentiation and yet represses adipocyte differentiation. At the cellular level, Msi2 affects indirectly and negatively on peroxisome proliferator-activated receptor (PPAR)γ, a key adipogenesis-related factor, thus inhibiting the adipogenic fate of MSCs. Indeed, Msi2 acts as a repressor of translation, by binding the 3′UTR of the target mRNA, particularly (G/A) U1-3 (AGU) motifs, to prevent poly-A binding protein from entering the extension initiation complex. Thus, Msi2 is one of the regulators of MSCs commitment, as well as a key factor in maintaining MSCs balance differentiation between adipogenesis and osteoblastogenesis (Suo et al., 2022).

In osteoclast precursors, Msi2 is the predominant isoform and, during osteoclast differentiation, its expression is upregulated by receptor activator of NF-κB ligand (RANKL) (Fujiwara et al., 2016). In these cells, Msi2 regulates Notch2 activation and hairy and enhancer of split 1 (Hes1) and knocking down of Msi2 attenuated Notch2/Hes1activation in pre-osteoclasts and mature osteoclasts. Therefore, the activation of Notch2 by Msi2 lead to the activation of NF-κB and nuclear factor of activated T cells 1 (NFATc1) influencing osteoclastogenesis (Fujiwara et al., 2016).

### 3.2 PUM1 and PUM2

PUMILIO1 (PUM1) and PUMILIO2 (PUM2) are eukaryotic RBPs belonging to a well-conserved RBPs PUMILIO (PUM) family, which has been described as regulators of the MSCs differentiation process (Spassov and Jurecic, 2003). Experiments on zebrafish and mammals have demonstrated that PUM1 is involved in the MSCs proliferation, while PUM2 in the regulation of fate determination of bone marrow-derived MSCs. *In vitro* experiments performed in MSCs with *PUM1* known-down have shown a reduction in the proliferation ability of MSCs, yet remaining stable in MSCs with *PUM2* ^*−/−*^ *.* In contrast, the depletion of *PUM2* impaired the capacity of MSCs to differentiate into the adipogenic lineage, by enhancing osteogenesis. At the molecular level, both RBPs PUM1 and PUM2 bind to the 3′UTRs of mRNAs; in particular, PUM2 acts by repressing the translation of a master regulator of MSC osteogenesis, distal-less homeobox 5 (DLX5), a known inhibitor of *PPARγ* expression (Shigunov et al., 2012; Lee et al., 2020).

These findings highlight the role of PUM2 as a strong post-transcriptional regulator in the fate determination of MSCs, pivotal to maintaining the physiological balance between osteogenic and adipogenic differentiation.

### 3.3 SAMD4

A novel osteogenic regulator, the sterile alpha motif domain containing protein 4 (SAMD4), has been identified in *Drosophila Melanogaster*. SAMD4 is an RBP with the role of protein translational repressor. It is mostly involved in the regulation of post-transcription events, including mRNA stability, and translational repression during early bone embryonic development (Wang and Zhang, 2023). *In vivo* experiments in mice (Niu et al., 2017) with a depletion of the *Samd4* gene, have shown profound defects in both skeleton development and bone mass; compared to WT mice, KO *Samd4* mice were characterized by impaired bone ossification and mineralization. Additionally, by *in vitro* experiments, the authors showed that SAMD4 is also involved in osteoblastogenesis. Primary osteoblasts collected from both KO *Samd4,* cultured under osteoblast differentiation medium, showed impaired of both early and late osteoblast differentiation when compared to those from WT mice. SAMD4 acts by inhibiting the translation of Mitogen-inducible gene 6 (Mig6) protein, a non-kinase scaffolding adaptor strongly expressed in osteoblasts, through binding the 3′UTR (Staal et al., 2014; Wang and Zhang, 2023).

Altogether, these studies have contributed to identifying the mammalian SAMD4 as a key regulator of skeletal development and as well as a regulator of osteogenesis.

### 3.4 CPEB4

Cytoplasmic polyadenylation element binding proteins (CPEBs) are multifunctional RBPs implicated in various biological processes and in the pathogenesis of certain diseases. Cpeb4, in particular, has been reported to be upregulated during osteoclast differentiation by regulating target mRNA translation in the cytoplasm (Arasaki et al., 2020). The authors speculate on a possible role of Cpeb4 in the repression of the translation of target mRNAs that inhibit osteoclast differentiation.

### 3.5 QKI

Quaking (QKI) is a member of the signal transduction and RNA activators (STAR) family, which has distinct functions in RNA metabolism with relevant impacts on cellular differentiation (Neumann et al., 2022). QKI has been shown to play a critical role in the regulation of osteoclastogenesis in mice with normal physiology and bone-associated pathology; indeed, QKI deficiency fostered OC differentiation and impaired bone metabolic balance. In detail, QKI deficiency in the myeloid lineage promoted OC differentiation through the activation of NF-κB and MAPK pathways induced by RANKL (Du et al., 2020). Using *in vivo* and *in vitro* mouse models, QKI has been shown to affect bone mass by influencing the early fate of BMSCs, impairing osteogenic differentiation and promoting adipogenic differentiation via the Wnt pathway (Yan et al., 2023).

## 4 Role of RBPs in the bone pathologies

At the cellular level, the constant bone remodelling is tightly balanced. A deregulated bone homeostasis can lead to abnormal bone remodelling, resulting in bone pathologies, such as osteoporosis, and is related to the microenvironment preparation to bone cancer and bone metastasis.

### 4.1 RBPs in osteoporosis and bone aging

During cellular aging, changes that affect all physiological systems of bone cells can also lead to a dysregulation of RBPs, which can contribute to impaired bone composition and, thus a predisposition to osteoporosis (OP) (Kelaini et al., 2021).

OP is a chronic progressive bone disease accompanied by a high risk of fractures, as a result of a decrease of both bone mineral density and bone mass (Noh et al., 2020). OP is a common condition in woman aging: during the post-menopausal period, an increased rate of bone resorption compared to bone formation may be the result of a loss of oestrogen and androgens-and in both sexes (primary OP), the bone loss can be generated from a progressive loss of MSCs, which reduces the supply of osteoblast precursors (Aibar-Almazan et al., 2022). OP can also be secondary to specific medical conditions that can affect bone remodeling, the most common being glucocorticoid-induced OP. At high concentrations, glucocorticoids drastically reduce the number and activity of osteoblasts and osteocytes, thus affecting the rate of bone formation (Compston, 2018).

Regardless of the causes of OP, bone loss is often accompanied by adipose accumulation in the bone marrow. In line with this, evidence points out that both a progressive depletion of osteoclast precursors and an increase in adipogenesis contribute to the aetiology of OP (Savopoulos et al., 2011). Therefore, the balance between osteoblasts and adipocytes also plays a critical role in maintaining bone homeostasis (Muruganandan et al., 2018).

#### 4.1.1 HuR

Human antigen R (HuR) is a RBP that regulates several post-transcriptional processes, as well as cellular events, including senescence, differentiation, proliferation and apoptosis (Srikantan and Gorospe, 2012). HuR has been suggested as a new possible target to counteract OP progression. In an *in vivo* model of OP obtained with ovariectomized (OVX)-mice and in *in vitro* osteoblast precursor cell line derived from embryo mouse calvaria (MC3T3), the induction of over-expression of HuR promotes osteogenic differentiation, osteoblast mineralization and collagen synthesis, thus arresting OP progression. HuR has been identified as an upstream regulator of osteogenic differentiation pathways; indeed, its silencing inhibits the differentiation of MC3T3 cells into osteoblasts. HuR acts by targeting an important target of OP, the low-density lipoprotein receptor-related protein 6 (LPR6), a key regulator for skeletal development and bone homeostasis through the regulation of Wnt/β-catenin-related signalling. HuR binds the 3′UTR of LPR6 mRNA, thus promoting its stability and stimulating its translation. In this manner, LPR6 can induce the activation of the Wnt pathway, promoting osteogenic differentiation (Maeda et al., 2013; Li et al., 2014; Liu et al., 2022; Ren et al., 2022).

#### 4.1.2 Msi2

A recent study has shown the RBP Msi2 as a new target molecule for aging-induced OP treatment (Suo et al., 2022). As discussed above, in physiological conditions Msi2 has been described as an essential player to regulate the balance of MSCs commitment, by enhancing osteoblast differentiation and repressing adipocyte differentiation (Park et al., 2014). Upon aging, MSCs intensify senescence, with a leaning to differentiate into adipocytes instead of osteoblasts (Chen et al., 2016). In line with this, Suo et al. (2022) showed how Msi2 expression changed during aging. BMSCs isolated from old mice (24 months) showed a decrease in Msi2 expression levels when compared to younger mice (2 months). By performing *in vivo* experiments with a depletion in *Msi2*, the authors showed that *Msi2* ^*−/−*^ mice are characterized by a decreased bone mass and increased marrow adipocytes, which mimics what happens in aging-induced OP. Taken together, these experiments suggested that Msi2 could be one of the contributors to aging-induced OP, as a reduction of Msi2 levels leads to a shift from osteoblastogenesis to adipogenesis. For all these reasons, modulation of Msi2 could be beneficial for the treatment of aging-induced OP (Suo et al., 2022).

#### 4.1.3 PUM2

OP can also occur when the balance between osteo- and adipo-progenitor cells is altered (Liu et al., 2011). In line with this, an increase of adipogenesis in the bone marrow is strongly correlated to the decrease of bone mineral density, contributing to the development of OP. As described above, in bone physiology PUM2 is essential to maintain balance in the MSCs differentiation, and, for this reason, it could have a potential role in OP (Spassov and Jurecic, 2003). In this regard, Yoon et al. (2023), identified PUM2 as a potential therapeutic target for bone-related diseases, including OP. By performing *in vivo* experiments on a rat model of calvarial defects and on an OVX mice model of postmenopausal OP, the authors have observed that a KO of *PUM2* and systemic delivery of PUM2 siRNA prevented bone loss, promoting respectively bone regeneration and alleviation of OP symptoms. As described above, PUM2 acts as an important regulator for osteogenic differentiation of human MSCs, DLX5 (Heo et al., 2017). Therefore, *PUM2* could be used as a potential target gene to prevent or reduce OP progression, by modulating the osteogenesis activity of DLX5.

#### 4.1.4 Sam68

SRC associated in mitosis of 68 kDa (Sam68) is an RBP that belongs to the STAR family, which has been shown to be involved in different cellular events, including RNA splicing transcription, signal transduction, translation, cell cycle progression and apoptosis. At the molecular level, Sam68 contains proline-rich sequences and tyrosine-rich regions at the C-terminus, which can be target of several proteins containing SH2 and SH3 domains. Sam68 can be post-translationally modified, including serine/threonine phosphorylation, thus influencing its subcellular localization and interaction with RNAs (Bielli et al., 2011).

Several studies (Frisone et al., 2015; Fu et al., 2016) have reported that Sam68 is highly involved in tumorigenesis; however, Sam68 is also highly expressed in the bone of developing mouse embryos, suggesting its role in bone physiology (Wen et al., 2017). Staining experiments on mice embryos have shown a strong presence of Sam68 in the nucleus of both osteoblasts and osteoclasts; despite its high expression, in *in vivo* experiments *Sam68* ^*−/−*^ mice did not show skeleton abnormalities. However, other investigations (Richard et al., 2005; Sanchez-Jimenez and Sanchez-Margalet, 2013) indicated that Sam68 could be one of the potential targets for the treatment of age-related bone loss. *In vivo* experiments showed that the absence of *Sam68* protected from the development of age-related bone loss in aged mice (23 months old) when compared with *Sam68* ^*+/+*^ mice. The loss of *Sam68* guarantees a continuous generation of osteoblasts, in order to preserve bone mass. In addition, *Sam68* KO mice have revealed different roles of Sam68 in aging bone; Sam68 regulates the differentiation of bone marrow mesenchymal cells, promotes adipocyte differentiation and inhibits osteoblast differentiation (Richard et al., 2005; Sanchez-Jimenez and Sanchez-Margalet, 2013).

Experiments aimed to map the Sam68-binding sequence showed that Sam68 regulates and associates with mRNAs by binding their 3′UTRs and, as well as poly (adenosine) and the poly (uridine) nucleotide tails at the 3′ end of target mRNAs (Itoh et al., 2002).

#### 4.1.5 QKI

QKI is highly involved in the mechanism of MSCs cell fate decision and differentiation, specifically in the commitment of MSCs between osteogenesis and adipogenesis. In a mouse model of glucocorticoid-induced OP, it has been demonstrated that mice with *QKI*-deficient MSCs preserved bone mass, when compared to control (Du et al., 2020). By *in vitro* experiments, the authors showed that a KO of *QKI* enhanced osteogenic differentiation and suppressed adipogenic differentiation; conversely, its overexpression inhibited osteogenic differentiation and promoted adipogenic differentiation. QKI interact with several mRNAs (around 6,281), associated with MSC osteogenic differentiation pathways, in particular Wnt/β-catenin signalling. Indeed, QKI reduced osteogenic differentiation by suppressing the Wnt/β-catenin pathway, in particular through direct binding to Wnt mRNA, in both cytoplasm and nucleus (Du et al., 2020; Yan et al., 2023). These findings highlight that QKI is one of the key regulators of bone fat differentiation, thus a QKI targeting in MSCs could be a strategy for the treatment of bone diseases, including glucocorticoid-induced OP.

In Table 1 a summary of physiological and pathological roles of RBPs in bone cells is reported.

**TABLE 1:** Summary of physiological and pathological roles of RBPs in bone cells.

RBPs	Role in bone	Bone disease	Direct/indirect targets	References
*Msi2*	Osteoclastogenesis	Not present	Not reported	Fujiwara et al. (2016)
*PUM1*	MSCs proliferation	Not present	Not reported	Shigunov et al. (2012)
*SAMD4*	Regulation of osteogenesis	Not present	Mig6	Niu et al. (2017)
*CPEB4*	Osteoclasts differentiation	Not present	Not reported	Arasaki et al. (2020)
*QKI*	Osteoclastogenesis	Not present	NF-κB and MAPK pathways	Du et al. (2020)
			Wnt pathway	Yan et al. (2023)
*HuR*	Osteogenic differentiation	Osteoporosis	LRP6	Liu et al. (2022)
*Msi2*	Balance of MSCs commitment	Osteoporosis	PPARγ signalling	Suo et al. (2022)
*PUM2*	Fate determination of MSCs	Osteoporosis	DLX5	Yoon et al. (2023)
*Sam68*	MSCs differentiation	Age-related bone loss	Not reported	Richard et al. (2005)
*QKI*	MSCs cell fate decision and differentiation	Osteoporosis	NF-κB and MAPK pathways	Du et al. (2020)

## 5 Role of RBPs in bone cancer

Recent genomic sequencing studies in malignant tumours, including bone cancers, have revealed several genetic mutations and abnormal expression of RBPs, indicating a pivotal role of these proteins in the initiation, development and progression of cancer (Qin et al., 2020; Zhang et al., 2021).

Bone cancers represent a small percentage of malignant tumours worldwide, constituting about 5% of childhood cancers and less than 1% of all cancers in adults. Bone cancers include osteosarcoma (OS), Ewing’s sarcoma (ES), fibrosarcoma, malignant fibrous histiocytoma and chordoma (Ferguson and Turner, 2018).

### 5.1 RBPs in osteosarcoma

OS is the most common bone tumours that localizes mainly in the metaphysis of long bones. OS has a bimodal distribution of incidence among age groups: the first peak occurs in children and adolescents, while the second peak of incidence concerns adults over 65 years old. Worse outcomes are observed in older patients compared to paediatric counterparts, where the disease is often accompanied by a higher rate of metastatic relapse. At the molecular level, OS can be generated from alterations of several processes, including the differentiation of MSCs in mature osteoblasts and transformed osteoblast cells producing osteoid matrix (de Azevedo et al., 2020). Gene Ontology (GO) enrichment analysis of clinical datasets samples (containing three normal samples and 84 tumour samples) displayed that the expression of the majority of RBPs were downregulated in OS, showing a correlation with RNA catabolic process, endonuclease and catalytic activity, ribosome biogenesis and ribosomal subunits (Li B. et al., 2021). Among these deregulated RBPs, it has shown a lower mRNA expression of DEAD-box helicase (DDX) 21 and 24 and insulin-like growth factor 2 mRNA binding protein (IGF2BP) 2 in osteosarcoma cell lines, when compared to osteoblast cell lines. DDX21 and DDX24 are RBPs involved in ribosomal RNA biogenesis and transcription processes, while the RBP IFG2BP2 is implicated in osteoblast differentiation (Xi et al., 2014; Gao et al., 2023). Other findings identified the presence of 142 RBPs involved in OS, mainly enriched in RNA splicing, mRNA metabolic process and regulation of translation activity. Among these 142 RBPs, the authors constructed a prognostic model of 10 RBP genes (see Table 2) associated with the prognosis of OS. Some of them are tightly correlated to tumours, such as telomerase reverse transcriptase (TERT), a ribonucleoprotein polymerase that adds telomerase repeats TTAGG to maintain telomere ends. TERT is highly expressed in various cancers and it is associated with poor prognosis (Bell et al., 2020; Li T. et al., 2021); cytoplasmic polyadenylation element-binding protein 3 (CPEB)3, which inhibits proliferation and migration of tumour cells if overexpressed; ribosomal protein S27-like (RPS27L), a regulator of genome stability, and eukaryotic translation initiation factor 4E family member 3 (EIF4E3), with both were correlated with tumour suppression functions (Zhang et al., 2021).

**TABLE 2:** Summary of the roles of RBPs in bone cancer and metastasis.

RBPs	Role in bone	Bone disease	Direct/indirect targets	References
TERT	Prognostic value	Osteosarcoma	Not Reported	Zhang et al. (2021)
CPEB3				
RPS27L				
EIF4E3				
TLR				
Treg				
RPS29				
TDRD6				
NXT2				
RBM10	Tumour suppressor	Osteosarcoma	Bcl2, caspase-3, TNFα	Han et al. (2018)
RBM34	Prognostic value	Osteosarcoma	Not reported	Zhang et al. (2021), Zhang et al. (2023b)
	Therapeutic target		Not reported	
LARP4A/4B	Pro-tumorigenic functions	Osteosarcoma	Cyclins B1 and E2, Aurora B, and E2F1	Coleman et al. (2024)
PUM2	Tumour suppressor	Osteosarcoma	RhoA/Rock signalling	Hu et al. (2018)
DDX24, DDX21, WARS IGF2BP2	Therapuetic target	Osteosarcoma	Not reported	Li et al. (2021a)
IGF2BPs	Diagnostic and prognostic role	Ewing’s sarcoma	Not reported	van de Luijtgaarden et al. (2013)
RBM3	Stemness capacity of cancer cells	Bone metastasis	Wnt/β-catenin	Zhang et al. (2023a)
		Bone metastasis		Lee et al. (2017)

A link between RBM10 and OS has been reported in an *in vitro* study in U2OS cell line. RBM10 seems to act as a tumour suppressor in OS through the inhibition of cell growth, cell migration and invasion and is responsible of the induction of cell apoptosis by inhibiting Bcl-2, activating caspase-3, and producing TNFα (Han et al., 2018).

Using multi-omics data, a close association between RBM34 and several cancer types was demonstrated. In OS, RBM34 significantly promoted cell proliferation and migration, and knockdown of RBM34 increased the percentage of cells in G1 phase, suggesting that RBM34 could regulate the cell cycle and cell proliferation (Zhang W. et al., 2023).

La-Related Proteins (LARPs) are a superfamily of RBPs and several of them have been associated with cancer. LARP4A and LARP4B are highly expressed in OS tissue and the depletion of LARP4A and LARP4B in MG63, osteosarcoma cell line, reduces the formation of lung metastatic foci, indicating a pivotal role for these proteins in the promotion of metastatic colonization. It has been demonstrated that LARP4 proteins, besides the cytoplasm, are expressed also in mitochondrial fractions as well as in the nucleus in MG63 cells, supporting an essential role for these RBPs in mitochondrial function (Coleman et al., 2024). The authors suggest a possible crosstalk between energy production and LARP4 protein function at the level of mitochondrial membranes, and this is consistent with the capabilities of LARP4A and LARP4B to function in energy-intensive processes such as cellular proliferation, migration, and cancer development.

By COX regression analysis, it has been reported that DDX24, DDX21, WARS and IGF2BP2 could be prognostic factors in OS and in particular, WARS seems to be related to osteosarcoma immune infiltration (Li B. et al., 2021).

PUM2 expression in OS tissues is significantly decreased respect to normal adjacent tissues and overexpression of PUM2 inhibits OS progression via suppressing RhoA/Rock pathway. STARD13 was identified as a direct target of PUM2 the link between PUM2 and 3′UTR STARD13 enhance STARD13 mRNA stability and expression. PUM2 competitively binds the STARD13 3′UTR with miR-590-3p and miR-9. Induction of PUM2 overexpression on STARD13 expression was mitigated by overexpression of miR-590-3p or miR-9, whereas PUM2 inhibition was preserved by overexpression of either miR-590-3p or miR-9. PUM2 and STARD13 3′UTR inhibit migration, proliferation and stemness of osteosarcoma cells (Hu et al., 2018).

#### 5.1.1 RBPs interaction with non-coding RNA in osteosarcoma

RBPs can cooperate with non-coding RNA and regulates diseases and malignant tumours. In OS HuR binds to high-mobility group AT-hook 1 (HMGA1) and miR-142-3p binds to the 3′UTR of HMGA1 to promote OS cell proliferation and epithelial-mesenchymal transition (EMT) while reduce cell apoptosis at the same time (Que et al., 2023).

PUM2, typically attenuated in OS tissue, through partially and competitive binding to the STARD13 3′UTR with miR-590-3p and miR-9, exerts an inhibitory effect on OS progression (Hu et al., 2018).

QKI2, one of the isoforms of QKI downregulated in OS, shows a role in limiting OS cell progression by miR-17–92 cluster competitively binding to QKI2, thereby upregulating β-catenin expression in osteosarcoma cells (Yang et al., 2018).

CPEB1 has an altered activity in OS, affecting the translation of critical genes involved in tumour growth and progression. It has been demonstrated that miR-320a regulates the expression of CPEB1 by targeting the 3′UTR of CPEB1 directly. Wang et al. (2020) demonstrated that downregulated CPEB1 inhibits osteosarcoma cell proliferation ability and metastasis.

IGF2BP1, upregulated in OS cells, is target gene of miR-150 and was negatively modulated by miR-150. MSC-derived exosomes carrying miRNA-150 act on proliferation, migration, invasion, and induced apoptosis of OS cells by targeting the RBP IGF2BP1 (Xu et al., 2020).

Long non-coding RNA (lnc) XIST, a poor prognosis factor associated with malignant phenotypes in OS, has been shown to be linked to HuR as possible regulators of OS progression. Indeed, the silencing of HuR inhibits OS cell EMT proliferation and migration through argonaute RISC catalytic component (AGO) 2 in association with lncRNA XIST (Liu et al., 2021).

lncRNA double homeobox A pseudogene 10 (DUXAP10), overexpressed in OS tissues, promoted OS cells proliferation, migration, and invasion of OS cells and inhibited their apoptosis. Wang et al. (2022) reported a correlation between DUXAP10 and HuR; SOX18, identified as one target of DUXAP10, can act as downstream factor of DUXAP10 to finally promote OS cell progression.

lncRNA anti-differentiating non-coding RNA (DANCR) can target OS cells and regulates tumorigenesis and development by suppressing miR-149 to positively regulate the expression of MSI2, therefore promotes the occurrence, development and progression of osteosarcoma (Zhang W. et al., 2020).

The study of Zhang Y. et al. (2020) demonstrated that miR-129-5p can bind to the RBP LARP1 and lncRNA KCNQ1OT1 and in turn can inhibit the progression of cell proliferation, invasion, and drug resistance when KCNQ1OT1 was knockdown (Zhang Y. et al., 2020). The authors suggest that KCNQ1OT1 might be considered a new biomarker related to proliferation and drug resistance of osteosarcoma.

All these data analyses emphasize the presence of altered RBP levels in bone cancers, and therefore the possibility of exploring RBPs as new targets or prognostic factors for OS.

### 5.2 RBPs in Ewing’s sarcoma

Ewing’s sarcoma is a rare and aggressive type of bone and soft tissue cancer primarily affecting children and young adults for which only few prognostic markers have been identified (Riggi et al., 2021). The involvement of RBPs in ES progression has been reported, and several studies have suggested their potential significance in the disease. In particular, recent studies have shown that IGF2BPs are overexpressed in ES and contribute to its malignancy by enhancing the expression of oncogenic genes and promoting cell migration and metastasis (van de Luijtgaarden et al., 2013). Elevated expression of IGF2BPs, particularly IGF2BP1 and IGF2BP3, has been observed in ES tissues compared to normal tissues. This overexpression is associated with increased cell proliferation and survival, suggesting a potential oncogenic role. Studies have explored the potential clinical significance of IGF2BPs as diagnostic or prognostic markers in ES. Patients with high expression of IGF2BP3 show high aggressiveness of the disease and poor probability of survival. IGF2BP3 increases the capacity of ES cells to growth under anchorage-independent conditions migrate and metastasize at distal organs (Mancarella et al., 2018). Thus, targeting IGF2BPs may represent a therapeutic strategy for inhibiting tumour growth and improving treatment outcomes (Chen et al., 2021).

## 6 Role of RBPs in bone metastasis

Bone is also one of the recurrent sites for metastasis formation by a variety of solid tumours, as well as lung, breast, prostate, thyroid, kidney cancers and malignant melanoma. Bone metastases represent a secondary growth site for cancer cells that, from their original site of growth, spread to distant organs including bone. At this stage, tumour cells disseminate through complex mechanisms requiring the molecular coordination of several activities, including protrusion, chemotaxis, invasion and contractility (Coleman et al., 2020). In addition, cancer cells must adapt to various stressors and different environments in order to metastasize successfully. Thus, the plasticity of cancer cells, driven by epigenetic and transcriptional mechanisms, is necessary for cancer cells to metastasize (Bravo-Cordero et al., 2012). All these changes require tight regulation from multiple factors at several levels, including post-translation modifications and post-transcriptional RNA processing. RBPs play a principal role in influencing gene expression through post-transcriptional regulation. Particularly, RBPs that are aberrantly expressed in cancer regulate the expression levels of target RNAs related to cancer cells such as proliferation, migration, EMT, invasion and angiogenesis (Kang et al., 2020; Weisse et al., 2020).

RNA binding motif protein 3 (RBM3), a stress response protein that belongs to the glycine-rich RNA-binding protein family, plays a crucial role in cell cycle progression and mitosis. In particular, RBM3 has been shown to be involved in cell adaptation under stress situations, such as oxygen depletion and thermal shock (low temperature) (Kim et al., 2018; Yan et al., 2019).

An *in vitro* study, aimed to mimic prostatic cancer cells bone metastases, showed that RBM3 is able to attenuate stem-like properties of prostate cancer cell line, DU145, when co-cultured with osteoblasts. In line with this, overexpression of RBM3 leads to a high reduction of prostate cancer cells’ stemness capacity, thus suggesting a role for RBM3 as a stemness suppressor. Indeed, the authors observed that the protein levels of RBM3 were significantly downregulated in bone metastases, compared to bone tumours (Zhang S. et al., 2023). At the molecular level, RBM3 acts indirectly by inhibiting Wnt/β-catenin pathway activation, which is required for bone metastasis of prostate cancer cells, thus enhancing cellular migration and invasion. In conclusion, restoring the expression of RBM3, which results in suppression in the bone microenvironment, could be a possible beneficial therapeutic approach for inhibiting prostate cancer bone metastasis (Kaplan et al., 2021; Wang et al., 2021).

In *in vivo* experiments, HuR was shown to promote breast cancer bone metastasis. This finding was supported by the reduction of HuR-knockdown metastatic potential in MDA-MB-231 breast cancer cells. The metastatic capacity of breast carcinoma cells expressing HuR was related to the secretion of CC chemokine ligand 2 (CCL2), a small cytokine with pro-tumorigenic and angiogenic effects that has been implicated in various metastatic processes, including bone metastasis (Lee et al., 2017).

In Table 2 a summary of the roles of RBPs in bone cancer and metastasis is reported.

## 7 Target RBPs as therapeutic strategy in bone diseases

There is evidence that transcriptional control and many post-transcriptional events are deeply embedded in RBPs’ regulatory circuits that contribute to the maintenance of cellular homeostasis (Hogan et al., 2008). Consequently, RBPs have been widely used to study therapeutic strategies in several diseases, including bone pathologies (Cornelius et al., 2022). As discussed above, RBPs have unique domains to bind their target RNAs in a sequence- and structural conformation-dependent manner. This allows for the development of good strategies to target directly specific RBPs or RBP-RNA interactions, thus expanding therapies for bone diseases (Hong, 2017). Emerging RNA-based therapeutic potentials, such as nuclease-associated genome editing technologies, small interfering duplex RNAs or antisense oligonucleotides, can lead to the knockdown, degradation or overexpression of specific target RNAs or the modification of RNA sequences to prevent the binding of a specific RBP (Lieberman, 2018; Wu, 2020).

Experiments by Xu et al. (2018) demonstrated that targeting HuR could be used as a potential therapeutic approach for the treatment of OS. In human OS cell lines, the authors showed that knockdown of HuR suppresses cell migration, invasion and stemness; it also increases the sensitivity of OS cells to the chemotherapeutic drug adriamycin, which is used to treat OS. Guevara-Aguirre et al. (2011) demonstrated that low IGF2BP3 expression could protect bone cells from cancer. Indeed, high protein levels of IGF2BP3 correlate with malignant transformation of bone cells, promoting proliferation, drug resistance and metastasis. Moreover, *in vitro* experiments on ES cell lines (Mancarella et al., 2018) have shown that silencing IGF2BP3 drastically reduces cell migration and growth.

On the other hand, other studies (Delmore et al., 2011; Hong, 2017) have shown that downstream effectors of RBPs can be used as therapeutic targets.

One example is MYC, an important regulator of gene expression. MYC is a target of several RBPs, such as HuR and hnRNPH A1, promoting their stability and modulating cancer progression (Stine et al., 2015). Indeed, several studies (David et al., 2010; Roos et al., 2016; Carabet et al., 2018) have shown that the use of small molecule inhibitors, identified as MYC transcriptional repressors; indirectly suppress the oncogenic activity of RBPs.

Taken together, these studies highlight the use of inhibitory molecules or techniques that directly regulate RBP expression or indirectly inhibit RBP function, which may represent a possible approach to suppress the progression of bone cancer cells and, in general, for the treatment of bone diseases.

## 8 Concluding remarks

By regulating transcriptional and post-transcriptional events, RBPs emerge as crucial players in influencing gene expression and cell fate decisions during bone formation and maintenance processes. Nevertheless, several studies highlight the association between RBP alterations and various bone pathologies, including osteoporosis and bone neoplastic diseases. This connection suggests a potential diagnostic and therapeutic role for RBPs in these clinical contexts. From a therapeutic perspective, it is additionally important to consider that RBPs have disparate roles, cell-specific and age-related expression patterns. Therefore, understanding the intrinsic mechanisms of RBPs action associated with particular physiological processes and diseases in bone could be important to expand the understanding of RBPs interactions networks.

In conclusion, considering the large number of RBPs, whose specific function is still not completely understood, this review underscores the potential impact of RBPs as therapeutic targets and diagnostic markers in bone disease and, it also emphasizes the role of RBPs in physiological mechanisms in bone.
